# Work ability and associated factors among female ready-made garment workers in Bangladesh

**DOI:** 10.1371/journal.pone.0325309

**Published:** 2025-06-03

**Authors:** Fathema Tuz Zohra, Md. Abdullah Saeed Khan, Shahria Sattar

**Affiliations:** 1 Department of Biomedical Science and Engineering, World University of Bangladesh, Dhaka, Bangladesh; 2 Department of Biomedical Engineering, National Institute of Preventive and Social Medicine, Dhaka, Bangladesh; 3 Department of Occupational and Environmental Health, National Institute of Preventive and Social Medicine, Dhaka, Bangladesh; State University of Bangladesh, BANGLADESH

## Abstract

**Background:**

The ready-made garment industry plays a crucial role in Bangladesh’s economy and has made a significant contribution to womenempowerment by employing millions of female workers. The work ability, reflecting the productivity and well-being of a worker, has been less explored among female garment workers. This study aimed to explore this gap.

**Methods:**

This cross-sectional study was conducted among 395 female garment workers aged ≥18 years at a factory in Dhaka from August-December 2023. Work ability was assessed using the Work Ability Index (WAI). Sociodemographic and work-related factors associated with WAI scores were identified using linear regression analysis.

**Results:**

The average age (±SD) of the participants was 31.05 ± 6.71 years. The Work Ability Index scores among participants showed a good mean (±SD) score of 40.65 ± 2.71 [Range: 7–49]. Younger age (p < 0.001), not having any children (p = 0.012), lower family income (<12,000 BDT) (p < 0.001), higher duration of job (p = 0.016) and working in the finishing section (p = 0.001) were associated with higher WAI scores. After adjustment, age (β = −0.06, 95% CI −0.10 to −0.01), income (β = −0.63, 95% CI −1.20 to −0.05), and working in finishing (adjusted β = 1.77, 95% CI: 0.32 to 3.21 compared to administration section) were independently associated with WAI scores.

**Conclusions:**

The study identified several factors that can be considered when planning interventions to enhance work ability and well-being of female garment workers.

## Introduction

Bangladesh is the second-largest RMG manufacturer and exporter country in the world [1]. The industry accounts for a remarkable 84.6% of the country’s total export earnings [2], making it the largest source of foreign earnings. Thereby, RMG has been a driving force for the nation’s economy and has contributed significantly to economic growth, employment generation, and poverty alleviation. With over 4 million workers employed in the sector [3], it has become a vital source of livelihood, particularly for women from underprivileged backgrounds.

The productivity and efficiency of the industry depend on the physical and mental fitness of the employees. Despite the success of the RMG industry, concerns have been raised regarding the occupational hazards and risks that garment workers often encounter [4]. Prolonged working hours [5], poor workplace ergonomics [6], exposure to harmful chemicals [7], and inadequate safety measures [8] have been identified as potential threats to the health and well-being of these workers. These adverse conditions can significantly impair their work ability, leading to a decline in productivity.

Work ability, a multidimensional construct that encompasses physical and mental dimensions, has been considered a marker of workers’ productivity and overall well-being [9]. Previous studies have identified various factors that can influence work ability among garment workers [10,11]. These factors include sociodemographic characteristics, work environment, work experience, job type, working hours, and workplace ergonomics, among others [10]. For instance, a study conducted by Kaewboonchoo *et al.* in four Southeast Asian countries found that older age, lack of physical exercise, lower income, and poor ergonomic workplace structure were associated with reduced work ability among textile workers [10]. On the other hand, in Indonesia, a study highlighted the reduced work performance associated with low work ability [12]. Poor work ability can lead to increased occurrences of musculoskeletal problems among garment workers [11], resulting in a further decrease in working performance. Therefore, the work ability of ready-made garment workers needs to be assessed and addressed to ensure maximum productivity with minimum risk to their health and well-being.

In Bangladesh, nearly 58% of ready-made garment workers are female [13], and they have to bear the double workload of both factories and families. Therefore, they are more vulnerable to work-related stress and fatigue, which, in turn, can cause reduced work ability [14,15]. Despite the significance of this issue, there is a paucity of research exploring the work ability of female garment workers and the associated factors in Bangladesh. This research gap represents an area which can inform on areas for improving the female garment workers’ well-being and productivity. Hence, this study was aimed.

## Materials and methods

### Study design, population, and period

This cross-sectional study was carried out among female garment workers aged 18 years and above in a ready-made garment factory located at Dokshinkhan, Dhaka, Bangladesh, between August to December 2023 with data collection taking place in the month of September. Garment workers who were absent on the day of data collection or were diagnosed with major mental disorders and those who were unwilling to participate in the study were excluded. A total of 395 female garment workers were purposively selected during the data collection period ensuring inclusion of workers from five sections of the garment factory: administration, finishing, linking, sewing and production & quality control.

### Data collection instrument and procedure

A semi-structured interviewer-administered questionnaire was used for data collection. The questionnaire was first pretested on a sample of 40 female garment workers in another garment factory located at Mirpur, Dhaka. After making a few adjustments, the questionnaire was finalized for data collection in the field. The questionnaire comprised sections on sociodemographic information, work-related factors, and work ability assessed by the Work Ability Index (WAI) [16]. Data was collected by face-to-face interviews of the respondents at their residential locations after working hours. The purpose of the study was explained and informed written consent was obtained before the interviews.

### Outcome and explanatory variables

The outcome variable was work ability of the participants. Work ability was defined as the ability to work [10] as determine by the WAI scale. However, as WAI incorporates the physical, mental and social context in scoring a worker’s ability to work, we were interested in exploring sociodemographic and work-related factors as explanatory variables. The sociodemographic explanatory factors included age, educational qualification, marital status, family type, number of children, and monthly family income. The work-related factors were duration of job (in years), work section, work hours per day, and extra hours worked per day.

### Work ability index and scoring

The Work Ability Index (WAI) [16] was utilized to evaluate the working ability of female garment workers. The WAI is a tool developed by the Finnish Institute of Occupational Health to assess an individual’s capacity to work, considering health and job demands [17]. It includes 7 items which capture physical, and mental states influencing work performance and sustainability. The items include- current work ability compared to the best in their lifetime (item 1), work ability in relation to job demands (item 2), the number of current disease groups diagnosed by a doctor (item 3), estimated work impairment due to diseases (item 4), sick leave taken in the past year (item 5); personal forecast of work ability two years from now (item 6); and mental resources concerning life in general, both at work and during leisure time (item 7). The first item gets the personal assessment of an individual on his/her ability to work scored on a 11-point (0–10) numeric scale. The second item takes a person’s assessment of their ability to work with respect to the physical and mental demands. The answers are taken on a 5-point Likert scale- very good (score: 5), rather good (4), moderate (3), rather poor (2) and very poor (1). These scores are then combined to get a range between 2–10. The third item determines the current disease(s) a person has from a list of 14 disease categories. For each disease (if present) a score of 2 (two) is given for “own opinion” and 1 (one) for “physician’s diagnosis”. Therefore, the total possible score for this item is 0–28. However, in the present study score ranged between 0–7 with participant’s answer being limited to “physician’s diagnosis” or “no”. The fourth item queried the personal assessment of work impairment due to diseases with scores ranging between 1 (complete inability to work) to 6 (no hindrance to work). The fifth item asked about illnesses experienced within last year which left the person unable to work for maximum 9 days (score: 4), 10–24 days (3), 25–99 days (2) and 100–354 days (1). A score of 5 was given if the person was able to work. The sixth item assessed the personal forecast of work ability for next two years with the following options: unlikely (1), not certain (4) and relatively certain (7). The last (seventh) item had three questions which explored mental resources concerning life in general, both at work and during leisure time. The three questions asked a person if considering the last three months, if he/she enjoyed regular daily activities (Q1), been active and alert (Q2) and felt him/herself full of hope about the future (Q3). Each questions had following answers: often (score: 4), rather often (3), sometimes (2), rather seldom (1) and never (0). The scores of these questions were then added to get the raw item 7 score.

Before calculating the total score by adding the scores of each item, the score for items 2, 3 and 7 were converted using specific scoring rules [17] outlined below. Item no 2: for physically demanding work, scores related to physical demands (rated 3–5) were multiplied by 1.5, while scores for mental demands (rated 1–2) were multiplied by 0.5. For mentally demanding work, the reverse were applied—physical demand scores (1–2) were multiplied by 0.5, and mental demand scores (3–5) by 1.5. If the job is both physically and mentally demanding, scores remain unchanged. In this study work in all sections of the garments factor except administration was considered physically demanding. Working in the administration was more mentally demanding than physical. For item 3, the number of physician-diagnosed diseases affects the score: no disease scores 7, one disease score 5, two diseases 4, three diseases 3, four diseases 2, and five or more diseases score 1. For item no 7, the total sum was converted as follows: a sum of 0–3 equals 1 point, 4–6 equals 2 points, 7–9 equals 3 points, and 10–12 equals 4 points. For example, if the individual scores 3, 4, and 3 on the three questions (totaling 10), the final score for item 7 is 4. The total scores ranged from 7 to 49 with a higher score indicating higher work ability of the person. The WAI tool has been documented to have good internal and predictive validity, as well as reliability [16].

### Statistical analysis

Data analysis was performed using R Studio Version 2024.04.1. Data cleaning was done through a thorough check and correction of any mis-entry and miscoding. There were no missing values or outliers in the data. Normality of continuous data was checked using Shapiro-Wilk test and Histogram with a normal curve. Descriptive statistics were calculated and presented in frequency (percentage) for categorical variables and mean (±standard deviation) for continuous variables. Pearson’s correlation, Welch two samples t-test and one-way analysis of variance (ANOVA) were used to test mean WAI scores across different categories of participant characteristics. Univariate and multivariable linear regression analysis was done to explore factors independently associated with WAI scores. A p-value <0.05 was considered statistically significant.

### Ethical considerations

The study protocol was approved by the Institutional Review Board of NIPSOM (Memo no: NIPSOM/IRB/2023/06). All procedures were conducted following the guidelines of the Declaration of Helsinki. Informed written consent was obtained from all participants after explaining the study objectives and assuring confidentiality and voluntary participation.

## Results

Table 1 presents the sociodemographic characteristics and work-related features of the 395 female ready-made garment workers who participated in the study. The mean age was 31.05 ± 6.71 years. Around 46% were aged 21–30, and 53% had primary education. The majority (78%) were married and lived in nuclear families (78%). Most participants had up to 2 children (72%) and a monthly family income ≥12,000 BDT (62%). Regarding work experience, 66% had 1–5 years, and one-third worked in production/quality control or sewing sections. Most (88%) worked 8 hours, while 97% reported working 2 extra hours.

**Table 1:** Sociodemographic characteristics and work-related features of the respondents.

Characteristic	N = 3951
Age (years)	31.05 (6.71)
Age categories	
<= 20	17 (4.30)
21-30	181 (45.82)
31-40	163 (41.27)
>40	34 (8.61)
Educational Qualification	
Illiterate	15 (3.80)
Can sign only	102 (25.82)
Primary	211 (53.42)
Secondary	59 (14.94)
Higher Secondary	8 (2.03)
Marital Status	
Married	309 (78.23)
Divorced	42 (10.63)
Widow	25 (6.33)
Unmarried	10 (2.53)
Separated	9 (2.28)
Family Type	
Nuclear	307 (77.72)
Joint	88 (22.28)
Number of Children	
Up to 2	286 (72.41)
> 2	77 (19.49)
None	32 (8.10)
Monthly Family Income (BDT)	
>=12000	245 (62.03)
<12000	150 (37.97)
Work Experience (years)	
1–5	259 (65.57)
6–8	109 (27.59)
>10	27 (6.84)
Work Section	
Production and quality control	132 (33.42)
Sewing	132 (33.42)
Linking	76 (19.24)
Finishing	36 (9.11)
Administration	19 (4.81)
Work hours	
8	349 (88.35)
>8	46 (11.65)
Extra hours worked	
2 hours	385 (97.47)
> 2 hours	10 (2.53)
1Mean (SD); n (%)	

**Fig 1:**
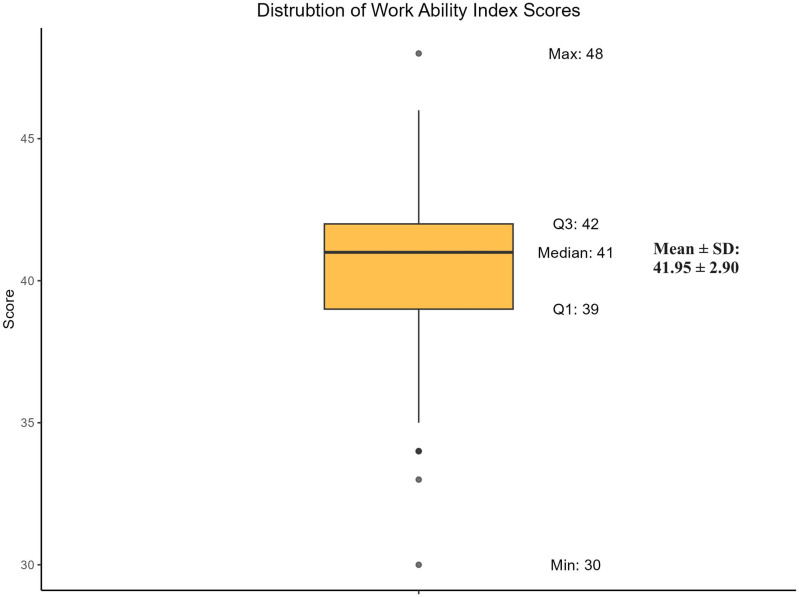
Distribution of Work Ability Index among participants.

Participants had a mean (±SD) Work Ability Index (WAI) score of 40.65 ± 2.71 and a median score of 41 (IQR: 39–42). The minimum and maximum scores were 30 and 48, respectively (Fig 1).

**Fig 2:**
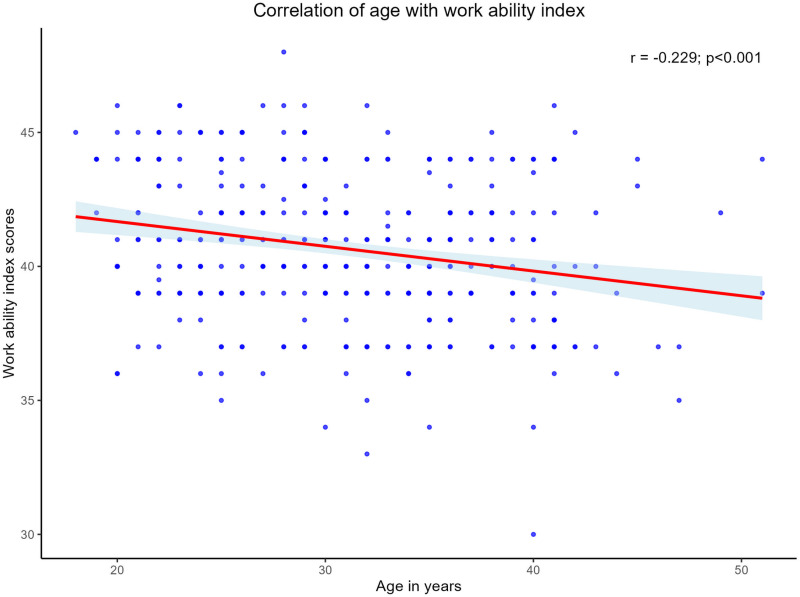
Correlation of WAI with age of the participants.

Analysis of the relationship between age and WAI scores revealed a negative correlation, as illustrated in Fig 2. The scatter plot with fitted regression line demonstrates a gradual decline in work ability scores as age increases, with notable individual variations.

Table 2 presents the distribution of WAI scores across various participant characteristics. The mean WAI score was significantly higher among those without children (41.95 ± 2.90) compared to those with children (40.54 ± 2.66, p = 0.012). Workers with lower income (<12,000 BDT) showed higher mean WAI scores (41.31 ± 2.99) than those with higher income (40.25 ± 2.44, p < 0.001). The work section was also significantly associated with WAI scores (p = 0.001), with finishing section workers showing the highest mean score (41.78 ± 2.58) and sewing section workers the lowest (39.96 ± 2.81). Workers with less experience (≤10 years) demonstrated significantly higher mean WAI scores compared to those with more experience (40.73 ± 2.73 vs 39.61 ± 2.17, p = 0.016).

**Table 2:** Work ability index scores by various participant characteristics.

Characteristics	Work Ability Index (mean ± SD)	p-value
**Education**		
Uneducated	40.24 ± 2.83	0.058^1^
Educated	40.83 ± 2.64	
**Marital Status**		
Married	40.62 ± 2.68	0.619^1^
Single^3^	40.78 ± 2.80	
**Family Size**		
Nuclear	40.53 ± 2.65	0.101^1^
Joint	41.09 ± 2.87	
**Any children**		
None	41.95 ± 2.90	**0.012** ^**1**^
One or more	40.54 ± 2.66	
**Income category (BDT)**		
<12000	41.31 ± 2.99	**<0.001** ^**1**^
≥ 12000	40.25 ± 2.44	
**Duration of job (years)**		
Up to 10	40.73 ± 2.73	**0.016** ^**1**^
>10	39.61 ± 2.17	
**Work section**		
Administration	40.16 ± 2.89	**0.001** ^**2**^
Finishing	41.78 ± 2.58	
Linking	41.00 ± 2.28	
Production and quality control	40.91 ± 2.69	
Sewing	39.96 ± 2.81	
**Work hours per day**		
Up to 8	40.68 ± 2.69	0.602^1^
More than 8	40.45 ± 2.87	
**Overtime duration (hours per day)**		
Up to 2	40.68 ± 2.71	0.274^1^
> 2	39.70 ± 2.63	

^1^Welch two samples t-test

^2^One-way ANOVA

^3^Includes: Never married, divorced, separated or widowed

Significant p-values were shown in bold

Table 3 shows the distribution of participants’ responses to the Work Ability Index questions. The majority (73%) rated their current work ability as 8 on a 10-point scale. Over half rated their work ability concerning physical (55%) and mental (56%) demands as moderate. Very few reported having diseases like musculoskeletal (1%), cardiovascular (0.25%), or other conditions that impaired their work ability (5%). Most of the participants enjoyed regular activities (61%), felt active and alert (70%), and were hopeful about the future (70%) during the last three months.

**Table 3:** Distribution of participants based on their responses to Work Ability Index questions.

Characteristic	N = 3951
Personal rating of work ability on a ten-point numeric scale	
Eighth	290 (73.42)
Nine	53 (13.42)
Seven	45 (11.39)
Six	7 (1.77)
Personal rating of work ability with respect to physical demand	
Moderate	216 (54.68)
Rather good	175 (44.30)
Very good	4 (1.01)
Personal rating of work ability with respect to mental demands	
Moderate	221 (55.95)
Rather good	171 (43.29)
Very good	3 (0.76)
Current diseases	
Musculoskeletal disease	
Absent	391 (98.99)
Present	4 (1.01)
Cardiovascular disease	
Absent	394 (99.75)
Present	1 (0.25)
Neurological or sensory disease	
Absent	389 (98.48)
Present	6 (1.52)
Digestive disease/condition	
Absent	391 (98.99)
Present	4 (1.01)
Genitourinary disease	
Absent	390 (98.73)
Present	5 (1.27)
Skin disease	
Absent	392 (99.24)
Present	3 (0.76)
Endocrine or metabolic disease	
Absent	393 (99.49)
Present	2 (0.51)
Estimated work impairment due to diseases	
No hindrance/no disease	374 (94.68)
Able to do, but it causes some symptoms	21 (5.32)
Number of whole days off taken during the last 12 months	
None	389 (98.48)
Maximum nine days	6 (1.52)
Personal estimation of whether she can work for the next two years in her current job	
Not certain	233 (58.99)
Relatively certain	122 (30.89)
Unlikely	40 (10.13)
Mental capacities	
Enjoyed regular activities during the last three months	
Rather often	242 (61.27)
Often	135 (34.18)
Sometimes	18 (4.56)
Have been active and alert during the last three months	
Rather often	277 (70.13)
Often	105 (26.58)
Sometimes	13 (3.29)
Felt full of hope about the future during the last three months	
Rather often	278 (70.38)
Often	106 (26.84)
Sometimes	11 (2.78)
1n (%)	

Multiple linear regression analysis revealed several independent predictors of Work Ability Index scores (Table 4). Age showed a significant negative association with WAI scores in both univariate (β = −0.09, 95% CI: −0.13 to −0.05) and multivariate analyses (β = −0.06, 95% CI: −0.10 to −0.01). Higher income (≥12,000 BDT) was associated with lower WAI scores (adjusted β = −0.63, 95% CI: −1.20 to −0.05). Workers in the finishing section showed significantly higher WAI scores compared to the administration section (adjusted β = 1.77, 95% CI: 0.32 to 3.21). Other work-related factors, including education status, marital status, and working hours, did not show significant associations with WAI scores in the adjusted analysis.

**Table 4:** Factors associated with work ability among participants.

	Univariate			Multivariate		
Characteristic	Beta	95% CI1	p-value	Beta	95% CI1	p-value
Age (years)	−0.09	−0.13 to −0.05	**<0.001**	−0.06	−0.10 to −0.01	**0.009**
**Education status**						
Educated	—	—		—	—	
Uneducated	−0.58	−1.2 to 0.00	0.051	−0.22	−0.81 to 0.36	0.451
**Marital Status**						
Married	—	—		—	—	
Single	0.17	−0.48 to 0.82	0.610	−0.02	−0.66 to 0.62	0.948
**Family Size**						
Nuclear	—	—		—	—	
Joint	0.56	−0.08 to 1.2	0.085	0.61	−0.02 to 1.24	0.058
**Any Children**						
One or more	—	—		—	—	
None	1.4	0.44 to 2.4	**0.004**	0.81	−0.17 to 1.79	0.106
**Income Category (BDT)**						
<12000	—	—		—	—	
>=12000	−1.1	−1.6 to −0.52	**<0.001**	−0.63	−1.20 to −0.05	**0.032**
**Work Experience (years)**						
>10	—	—		—	—	
Up to 10	1.1	0.06 to 2.2	**0.038**	0.86	−0.23 to 1.96	0.122
**Working section**						
Administration	—	—		—	—	
Finishing	1.6	0.14 to 3.1	**0.032**	1.77	0.32 to 3.21	**0.017**
Linking	0.84	−0.50 to 2.2	0.217	0.76	−0.56 to 2.08	0.258
Production and quality control	0.75	−0.53 to 2.0	0.250	0.96	−0.31 to 2.24	0.139
Sewing	−0.20	−1.5 to 1.1	0.764	0.00	−1.28 to 1.28	0.997
**Work hours**						
>8	—	—		—	—	
8	0.23	−0.60 to 1.1	0.581	0.70	−0.16 to 1.56	0.112
**Extra hours worked**						
> 2 hours	—	—		—	—	
2 hours	0.98	−0.73 to 2.7	0.260	1.09	−0.63 to 2.81	0.213
1CI = Confidence Interval; Significant p-values were shown in bold						

## Discussion

The findings of this study provide valuable insights into the work ability of female garment workers in a ready-made garment factory in Bangladesh and the associated factors. The Work Ability Index scores showed a consistent distribution with most workers maintaining moderate to good levels of work ability, as evidenced by the median score of 41 and interquartile range of 39–42. These results align with previous studies conducted in Indonesia, Malaysia, Thailand, and Vietnam, where more than 70% of participants showed good to excellent work ability [10].

Notably, several sociodemographic and work-related factors were found to be significantly associated with work ability. Age showed a significant negative correlation with work ability scores, while having no children, lower family income, and working in the finishing section were positively associated with higher work ability scores. These findings partially align with existing literature [10] that has identified age as a key determinant of work ability, though some associations differ from previous findings.

The negative correlation between age and work ability can be attributed to the generally better physical and mental health status of younger individuals, which enables them to cope more effectively with the demands of their work [18,19]. Additionally, the lack of childcare responsibilities among women without children may contribute to their enhanced work ability by reducing the burden of domestic and caregiving tasks, as reflected in their significantly higher mean WAI scores (41.95 ± 2.90 vs 40.54 ± 2.66).

Interestingly, the study found that workers in the finishing section had significantly higher work ability scores (41.78 ± 2.58) compared to other sections, particularly the sewing section (39.96 ± 2.81). This finding could be attributed to the nature of the tasks and work environment in the finishing section, which may involve less physically demanding tasks or better ergonomic conditions compared to other sections. Moreover, the administration section has to deal with significant mental stress associated with the management of a large workforce in the garments, potentially affecting their work ability scores.

Unlike the study conducted by Kaewboonchoo et al. [10], we found that lower family income is associated with higher work ability scores, as evidenced by both univariate and multivariate analyses. This counterintuitive finding could be explained by the fact that the WAI focuses mainly on workers’ perception of their work ability and their physical conditions. Younger workers were more likely to be physically fit and might have rated well in their perception of work ability despite having a lower salary structure than the older ones. Then again, the lower salary could be due to younger and able people’s entry into the different sections as helpers rather than operators.

Female garment workers often have to work for 12 hours or more per day in a crowded environment without separate washrooms, designated facilities for menstrual hygiene, or health insurance [20]. On top of that, workplace harassment from male colleagues and managers is a frequent problem creating high mental stress in the workplace. Therefore, while the overall WAI score of these female garment workers appears high, there is substantial room for improvement, particularly in addressing the physical and mental demands and hazards associated with their work.

Furthermore, the prevalence of diseases and conditions that could impair work ability was relatively low among the participants. This finding may be attributed to the relatively young age of the study population, as well as the diagnostic assessment criteria for the diseases used by the physicians. Self-reported symptoms by the garments workers have been found higher in a previous study [21].

### Strengths and limitations

The study was one of the earliest attempts to explore work ability among female garment workers in Bangladesh, a public health issue crucial for improving the well-being and promoting sustainable development in the garment industry. While this study provides valuable insights, it had some limitations. First, the cross-sectional nature of the study precludes the establishment of causal relationships between the identified factors and work ability. Longitudinal studies are needed to examine the temporal relationships and potential causal pathways. However, this study provides a valuable exploratory starting point for future research. Second, the study was conducted in a single garment factory, which may limit the generalizability of the findings to other garment factories or regions within Bangladesh. Future research should consider a more diverse and representative sample of garment workers from multiple factories and locations. Third, the study relied on self-reported data, which may be subject to recall or social desirability bias. Fourth, in the process of making the study approach simple and subjective it failed to include occupational exposure variables like physical workload, repetition motion frequency, rest-break policies and social support factors like colleague relationships and management attitudes missing important contextual elements. Hence futures studies should consider incorporating objective measures of work ability, environment, and work-related factors and should include evaluators of social factors to make more comprehensive assessment of work ability determinants.

## Conclusion

The study shows that female garment workers generally have good work ability, with only one-fifth of them having excellent work ability. The findings highlight the importance of addressing age, income and job type in efforts to promote and maintain good work ability among this workforce. Hence, we have the following recommendations for garments authorities and policy makers:

- Design tasks and provide training tailored to different age groups to enhance productivity and reduce strain, especially among older workers.
- Ensure fair and adequate compensation for all and ensure all wedge-groups are maintaining their work ability to a standard level through equitable workload distribution preventing work-related fatigue.
- Conduct regular assessments of each work section (e.g., sewing, finishing, packaging) to identify and mitigate physical or psychological demands that may reduce work ability.
- Focus resources on work sections identified as having lower average work ability, offering ergonomic improvements, health support, and workload management.

## Supporting information

S1 File Strobe checklist for cross-sectional study. (DOCX)

S2 File Dataset. (CSV)

S3 File Variable names and coding rules. (XLSX)
